# Electrophysiological adaptations of insect photoreceptors and their elementary responses to diurnal and nocturnal lifestyles

**DOI:** 10.1007/s00359-019-01392-8

**Published:** 2019-12-19

**Authors:** Roman V. Frolov, Irina I. Ignatova

**Affiliations:** Nano and Molecular Systems Research Unit, University of Oulu, Oulu, Finland

**Keywords:** Microvillar photoreceptors, Photoreceptor evolution, Quantum bumps, Nocturnal vision

## Abstract

Nocturnal vision in insects depends on the ability to reliably detect scarce photons. Nocturnal insects tend to have intrinsically more sensitive and larger rhabdomeres than diurnal species. However, large rhabdomeres have relatively high membrane capacitance (*C*_m_), which can strongly low-pass filter the voltage bumps, widening and attenuating them. To investigate the evolution of photoreceptor signaling under near dark, we recorded elementary current and voltage responses from a number of species in six insect orders. We found that the gain of phototransduction increased with *C*_m_, so that nocturnal species had relatively large and prolonged current bumps. Consequently, although the voltage bump amplitude correlated negatively with *C*_m_, the strength of the total voltage signal increased. Importantly, the background voltage noise decreased strongly with increasing *C*_m_, yielding a notable increase in signal-to-noise ratio for voltage bumps. A similar decrease in the background noise with increasing *C*_m_ was found in intracellular recordings in vivo. Morphological measurements of rhabdomeres were consistent with our *C*_m_ estimates. Our results indicate that the increased photoreceptor *C*_m_ in nocturnal insects is a major sensitivity-boosting and noise-suppressing adaptation. However, by requiring a compensatory increase in the gain of phototransduction, this adaptation comes at the expense of the signaling bandwidth.

## Introduction

Rhabdomeric photoreceptors in insect compound eyes respond to photons of light with quantum bumps, each caused by activation of a single microvillus. The amplitude and kinetics of the elementary light signal, the voltage bump, are determined by two factors, the gain of phototransduction and the gain of the membrane (Frolov 2019). Phototransduction gain is set by the depolarizing sodium and calcium charge influx through the light-activated transduction channels during the quantum bump and thus is directly proportional to the number of open light-activated channels. The membrane gain depends on the activation state of the entire channelome and is inversely proportional to the total number of open channels. Both the phototransduction and membrane gains change dynamically with light adaptation, albeit via different mechanisms, decreasing in brighter light to compress the signaling range and facilitate transmission of higher-frequency signals, and increasing in the dark to improve the reliability of single-photon detection (Juusola et al. 1994; Juusola and Hardie 2001; Frederiksen et al. 2008; Heras et al. 2018).

Photoreceptors of species evolutionarily adapted to different ecological and behavioral conditions possess dissimilar gain control mechanisms. For example, fast-flying day-active flies are characterized by relatively fast phototransduction, which is strongly and dynamically modulated by Ca^2+^ influx via TRP channels (Hardie and Minke 1992; Niemeyer et al. 1996; Reuss et al. 1997; Leung et al. 2000). Large K^+^ conductance gives such photoreceptors low input resistance and thus low membrane gain (Weckstrom et al. 1991). An example of the opposite development is given by photoreceptors of night-active cockroach *Periplaneta americana*, which are characterized by the relatively slow phototransduction culminating in the opening of mostly TRPL channels that have lower selectivity for Ca^2+^ than TRP (Saari et al. 2017). This results in large quantum bumps and a weak dependence of phototransduction on the external Ca^2+^ (Immonen et al. 2014b). Also, high membrane resistance at rest provides high gain (Heimonen et al. 2012).

A crucial factor modifying signal gain is membrane capacitance (*C*_m_). It enables low-pass filtering that slows membrane voltage response, attenuates elementary signals (Frolov 2019), and narrows the photoreceptor bandwidth (Frolov 2016). Membrane capacitance is proportional to the cell membrane area, to which the highly convoluted rhabdomere is expected to contribute heavily, and thus can serve as a useful measure of photoreceptor size (Frolov et al. 2018). There are two groups of insect species where increased *C*_m_ apparently represents evolutionary adaptations. Firstly, photoreceptors of nocturnal insects tend to have higher *C*_m_*and* higher absolute sensitivity than photoreceptors of diurnal insects (Frolov 2016). Secondly, the highly visual fast-flying species have evolved relatively large rhabdomeres to improve the signal-to-noise ratio (SNR) in daylight via an increase in the number of sampling units, the microvilli (Niven et al. 2007; Song and Juusola 2014). Apparently to offset the effects of high *C*_m_ on signal processing, such fast fliers have to additionally evolve very leaky photoreceptor membranes (Weckstrom et al. 1991).

As a result of evolutionary differences in gain control mechanisms, elementary voltage responses in photoreceptors of diurnal species are notably smaller than in the photoreceptors of nocturnal ones (Frederiksen et al. 2008; Honkanen et al. 2017). Such differences must have consequences for vision in the dark, which relies on the processing of discrete voltage bumps (Honkanen et al. 2014). For successful transmission across the first visual synapse, the voltage bump needs to significantly exceed the surrounding voltage fluctuations of the membrane noise, i.e., to have a sufficiently high SNR. However, considerations of cellular economy also suggest that the voltage bump signal should not be too high, to avoid the excessive metabolic costs associated with signal propagation and synaptic release. While the evolution of membrane gain mechanisms has been studied fairly well (Frolov et al. 2016; Laughlin and Weckström 1993), the evolution of phototransduction gain and its role in shaping elementary responses remain a largely unexplored question, mainly due to the scarcity of comparative voltage and especially current quantum bump data.

In this study, we analyzed elementary responses of photoreceptors in ten insect species from six orders, Blattodea, Diptera, Hemiptera, Lepidoptera, Orthoptera, and Phasmatodea, using patch clamp, and we also recorded from these and other species in vivo. The species we studied occupy dissimilar visual ecological niches and exhibit different behaviors. Data were recorded in current and voltage clamp modes, allowing direct comparison of voltage and quantum bumps from the same photoreceptors. We discovered several general trends, including complex variation of quantum bump properties with *C*_m_, and dependencies of membrane noise and voltage bump SNR on *C*_m_. These results can help explain not only the superior signal processing by nocturnal species in the dark, but also the previously reported differences in signal processing in relatively bright light.

## Methods

Animals used in experiments were either reared locally (cockroaches, fruit flies, blowflies), or purchased from suppliers (Blades Biological, Inc., UK: stick insects, lesser water boatman), or caught locally (water strider, water boatman). Butterfly *P. xuthus* was a gift of Prof. Kentaro Arikawa (Sokendai, Japan).

With the exception of *P. americana* and *D. melanogaster*, all species were maintained in the animal room under normal illumination conditions at room temperature (22–24 °C). *P. americana* and *D. melanogaster* were maintained in incubators under the reverse illumination conditions, and the recordings were performed during the subjective night (patch clamp) or during both day and night (intracellular experiments). No differences were observed between responses of *P. americana* photoreceptors obtained at different times of the day. The data were obtained from at least three specimens in each species (for *C. vicina*, *P. xuthus*, *H. illucens* and *P. schultei*), but in other cases from ten or more animals.

All electrophysiological recordings were performed from green-sensitive photoreceptors.

### Patch-clamp recordings

Experiments were performed in the interval between 10.00 and 17.00 hours. Retinal tissue was dissociated and whole-cell recordings were performed as described previously for different species (Frolov and Weckstrom 2014; Frolov et al. 2014, 2015, 2017; Frolov 2019; Immonen et al. 2014a). In brief, the eyes were removed under CO_2_ anesthesia and retinal tissue carefully scooped out of the cornea, and then cut onto small pieces. In the majority of preparations, dissociation of ommatidia was preceded by incubation of the retinal fragments in the bath solution (below) supplemented with 0.2 mg/ml collagenase type 2 (Worthington Biochemical Corp., Lakewood, NJ, USA) and 0.2 mg/ml pancreatin (Sigma-Aldrich) for 5–10 min at room temperature. In some species, e.g., *N. glauca*, enzymatic treatment was not necessary for dissociation by trituration.

Patch-clamp data were acquired using an Axopatch 1-D patch-clamp amplifier, Digidata 1550 digitizer, and pClamp 10 software (Axon Instruments/Molecular Devices, CA, USA). Patch electrodes were made from a thin-walled borosilicate glass (World Precision Instruments, Sarasota, FL, USA). Bath solution contained (in mM): 120 NaCl, 5 KCl, 4 MgCl_2_, 1.5 CaCl_2_, 10 N-Tris-(hydroxymethyl)-methyl-2-amino-ethanesulfonic acid (TES), 25 proline and 5 alanine, pH 7.15. Two different patch pipette solutions were used. In the earlier studies, we used a solution containing (in mM): 140 KCl, 10 TES, 2 MgCl_2_, 4 Mg-ATP, 0.4 Na-GTP and 1 NAD, pH 7.15 (with KOH). The second solution was used in later studies of *P. nivea* and *P. americana* with the purpose of suppressing the hyperpolarization-activated Cl^−^ current (Salmela et al. 2012). It contained (in mM): 100 K-gluconate, 40 KCl, 10 TES, 2 MgCl_2_, 4 Mg-ATP, 0.4 Na-GTP and 1 NAD, pH 7.15. Apart from the differences in liquid junction potential (LJP) and suppression of the Cl^−^ current, no physiological differences were found. The LJP was − 4 mV for the first solution and − 12 mV for the second solution; all voltage values in the article were corrected for the LJP. Resistance of patch electrodes varied from 4 to 9 MΩ. Series resistance was compensated by about 80%. Membrane capacitance (*C*_m_) was calculated from the total charge flowing during capacitive transients for voltage steps below resting potential (Fig. 3a). Input resistances were estimated from leak currents at voltages that did not elicit voltage-activated currents (usually < − 60 mV) as described previously (Frolov 2019).

Current bumps were recorded at holding potentials (HP) varying from − 74 to − 92 mV. (The variability is due to changes in recording protocols and bath solution over years of data acquisition.) Because the amplitude depends on the driving force, all values were consequently corrected as if they were recorded at an HP of − 82 mV by using a common reversal potential value of +10 mV (in between the reversal potentials for light-induced currents in *D. melanogaster* and *P. americana* (Reuss et al. 1997; Immonen et al. 2014b)).

Continuous dim green light emitted by an LED with a peak at 525 nm was used to stimulate photoreceptors. Stimulus intensity was attenuated with a series of neutral density filters (Kodak, New York, NY, USA). Only photoreceptors with stable resting potentials ≤ − 45 mV were used for analysis. Recordings were performed at room temperature (20–24 °C).

### Intracellular recordings

Preparation and in vivo intracellular single-electrode recordings were performed as described previously (Saari et al. 2017). Photoreceptor responses were recorded using microelectrodes (borosilicate or aluminosilicate glass; Harvard Apparatus) manufactured with a laser puller (P-2000; Sutter Instrument) and filled with 2 M KCl and 0.2 M KH_2_PO_4_ solution, pH 6.84, or 2 M CH_3_CO_2_K and 2 mM KCl, pH 6.5, depending on the species studied, to a final resistance of 100–170 MΩ. The reference electrode was placed through the left antenna (cockroach), or into the second eye (fruit flies), or in the insect’s thorax/abdomen (others). Signals were recorded with a single-electrode amplifier (SEC-05L; NPI). 100–500 ms current steps ranging from − 1 to 1 nA in various increments (usually 0.25 nA) were used.

Membrane capacitance was estimated from voltage responses to current injection (Fig. 3b).

### Imaging

TEM images of *C. vicina* retina were obtained at the Biocenter Oulu imaging facility using a previously described protocol (Frolov et al. 2017).

### Data analysis

During data analysis, the Shapiro–Wilk normality test was applied to data samples to determine if they could be analyzed using parametric statistical methods. As most of data samples passed the normality test, we presented all data as mean ± S.D. Spearman’s rank order correlation coefficient (SROCC, *ρ*) was used to analyze correlations. Throughout the text, (*n*) stands for experimental group size.

## Results

### Quantum bumps in patch-clamp experiments

**Fig. 1:**
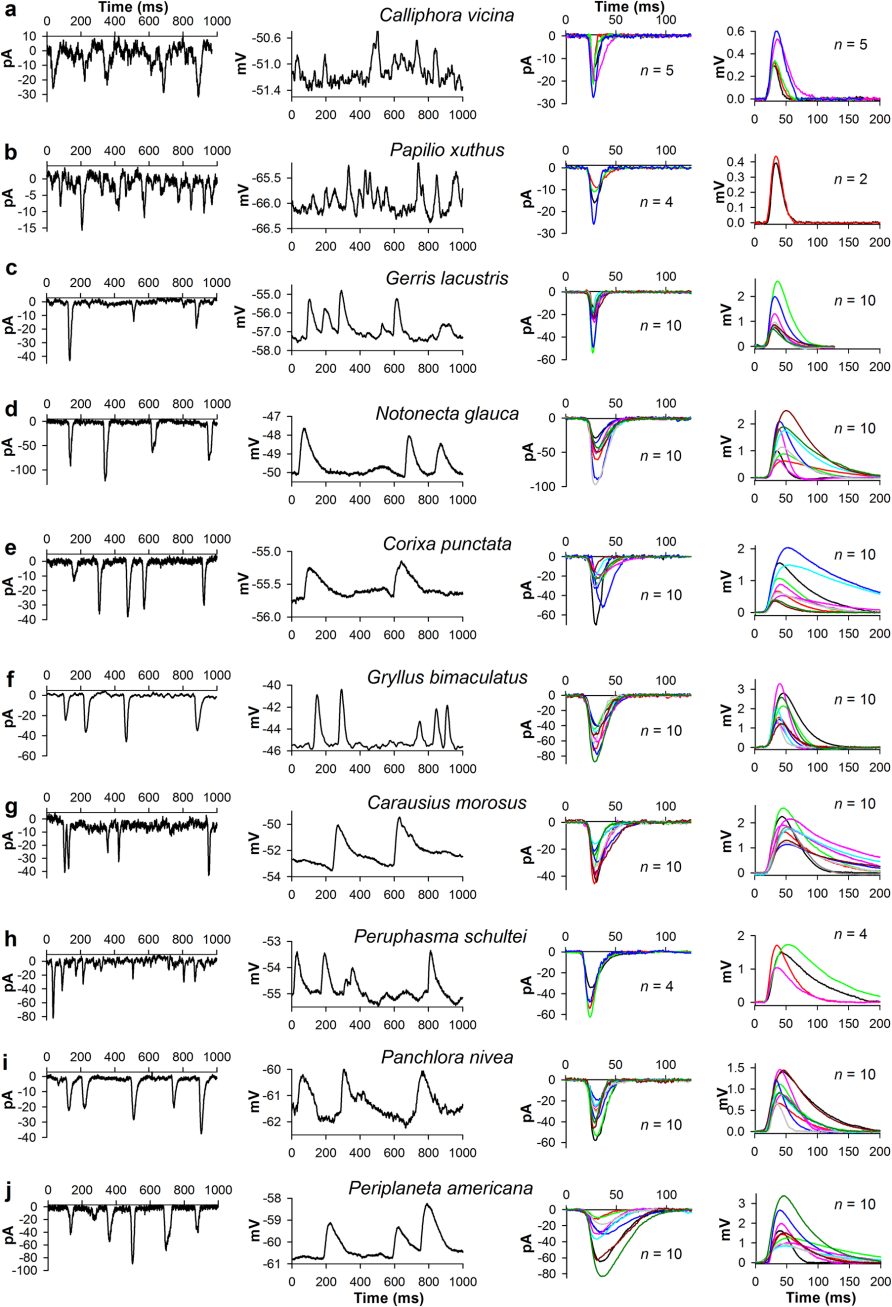
Comparison of elementary light responses. Each panel shows elementary responses from one species as indicated by labels in the center. Left and center-left subpanels: 1-s examples of current (left sub-panels, pA) and voltage (center-left subpanels, mV) traces containing isolated bumps evoked by constant dim light stimulation in voltage- and current-clamp recordings, respectively. Center-right and right subpanels: mean current and voltage bumps from different cells; notice different amplitude scales; to obtain mean bumps, 20–50 individual bumps in each photoreceptor were aligned by the rising phases and averaged; *n* stands for the number of photoreceptors; different colors designate different mean bumps

Patch-clamp recordings were performed from dark-adapted photoreceptors in dissociated ommatidia of ten species: blowfly *Calliphora vicina* (Diptera), butterfly *Papilio xuthus* (Lepidoptera), water strider *Gerris lacustris* (Hemiptera), water boatman *Notonecta glauca* (Hemiptera), lesser water boatman *Corixa punctata* (Hemiptera), cricket *Gryllus bimaculatus* (Orthoptera), stick insects *Carausius morosus* and *Peruphasma schultei* (Phasmatodea), and cockroaches *Panchlora nivea* and *Periplaneta americana* (Blattodea) (Fig. 1). The first three species are strictly diurnal; *N. glauca* and *C. punctata* can function in a wider illumination range (*C. punctata* is indicated as diurnal in Fig. 3d); the last five species are crepuscular or nocturnal.

Quantum bumps were recorded in both voltage- and current-clamp modes using low-intensity continuous light stimulation adjusted to elicit < 10 bumps s^−1^. The left subpanels of each panel in Fig. 1 show examples of current bump responses. Center-left subpanels show examples of voltage bump responses to continuous stimulation. Center-right and right subpanels demonstrate mean current and voltage bumps, respectively, obtained by aligning the rising phases of 10–50 individual bumps in each photoreceptor followed by averaging. In three out of ten species, the number of cells studied was below 10 as indicated. For all other species, the number of mean bumps shown was limited by ten for presentation purposes.

The current bumps in photoreceptors of three fast-moving diurnal insects, blowfly *C. vicina*, butterfly *P. xuthus* and water strider *G. lacustris*, appear to be smaller in size than the current bumps in other species (Fig. 1, center-right subpanels; Table 1, compare current bump amplitudes, half-widths and especially current bump integrals). Likewise, the blowfly and the butterfly were characterized by particularly small voltage bumps, all well below 1 mV, whereas voltage bumps in the water strider were bigger (Fig. 1, right subpanels). This difference is caused by very low input resistances of photoreceptors in *C. vicina* and *P. xuthus* compared to *G. lacustris* and other species (Table 1). A trend could be observed in the duration of voltage bumps, which were much wider in the crepuscular/nocturnal than in diurnal species (Fig. 1, center-left and right subpanels).

**Table 1:** Group-average electrophysiological parameters

Species	*C*_m_, pF	Input conductance, nS	Current bump, pA	Current bump half-width, ms	Current bump integral, pA × ms	Voltage bump, mV	Voltage bump integral, mV × ms	Noise s.d., mV
*C. vicina*	82 ± 42	9.5 ± 6.2	− 20 ± 4	6.8 ± 3.2	− 66 ± 63	0.5 ± 0.1	10 ± 5	0.16 ± 0.04
*P. xuthus*	127 ± 35	11.0 ± 4.2	− 16 ± 7	10.8 ± 2.1	− 244 ± 92	0.6 ± 0.03	8 ± 1	0.14 ± 0.01
*G. lacustris*	65 ± 19	1.7 ± 0.7	− 25 ± 13	6.4 ± 1.8	− 188 ± 98	1.5 ± 0.6	31 ± 22	0.23 ± 0.11
*N. glauca*	285 ± 124	5.2 ± 2.7	− 55 ± 20	16.5 ± 2.0	− 1026 ± 380	1.6 ± 0.6	77 ± 52	0.13 ± 0.04
*C. punctata*	419 ± 130	2.0 ± 2.0	− 28 ± 15	14.6 ± 4.3	− 481 ± 208	0.9 ± 0.5	76 ± 74	0.07 ± 0.03
*G. bimaculatus*	162 ± 94	6.1 ± 3.0	− 57 ± 16	16.8 ± 2.9	− 1005 ± 325	1.9 ± 0.6	70 ± 38	0.16 ± 0.03
*C. morosus*	196 ± 81	2.5 ± 1.0	− 31 ± 9	13.7 ± 3.6	− 468 ± 167	1.8 ± 0.4	151 ± 63	0.14 ± 0.05
*P. schultei*	227 ± 65	2.6 ± 0.6	− 41 ± 12	12.5 ± 1.8	− 699 ± 78	1.4 ± 0.2	97 ± 44	0.16 ± 0.05
*P. nivea*	265 ± 72	2.9 ± 1.6	− 36 ± 10	14.6 ± 3.5	− 492 ± 145	1.3 ± 0.5	77 ± 57	0.08 ± 0.02
*P. americana*	384 ± 156	2.1 ± 1.1	− 44 ± 19	27.8 ± 6.1	− 1164 ± 556	1.5 ± 0.7	121 ± 87	0.11 ± 0.04

The number of measurements for each group average are provided in legends to Figs. 4 and 5

**Fig. 2:**
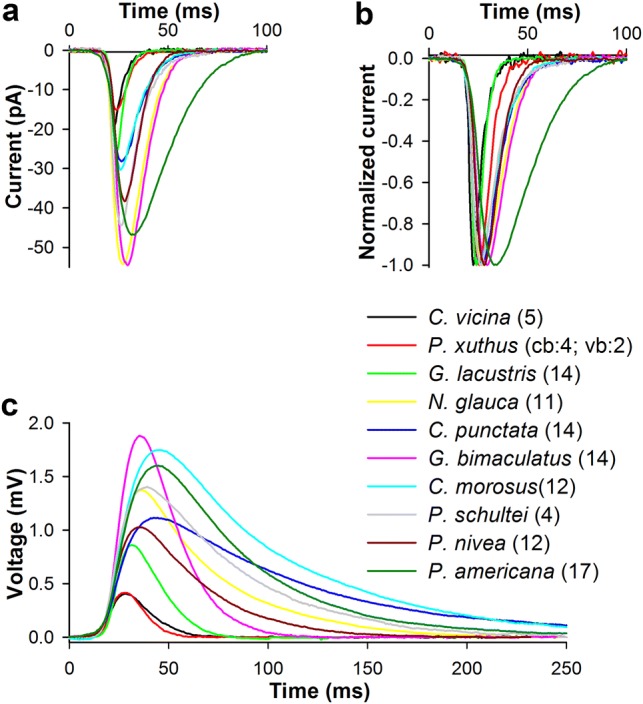
Group-average current and voltage bumps. **a** Group-average current bumps obtained by averaging mean bumps for each species, respectively. **b** Normalized group-average current bumps; the group-average bump amplitudes from **a** were used for normalizing. **c** Group-average voltage bumps; the plot was stretched to match the timescale of the current bump plot in **a**; in the legend for *P. xuthus*, “cb” stands for the current and “vb” the voltage bumps; numbers in parentheses indicate how many mean bumps were used to obtain each group average

Next we compared the group-average current and voltage bumps, which were obtained by averaging mean bumps from each photoreceptor in an experimental group (Fig. 2a–c). Current bumps from different species were characterized by dissimilar amplitudes and durations (Fig. 2a, b). The current bump is the fastest photoreceptor response, and it sets the upper boundary of the frequency response range. Therefore, the differences in the half-widths of the group-average current bumps (Fig. 2b, Table 1) predetermine the intrinsic differences in temporal resolution, with a provision for a further decrease in bump duration with light adaptation (Juusola and Hardie 2001). Likewise, the differences in the total depolarizing charge passed into the cell during the current bump reflect the evolutionary differences in the gain of phototransduction. As expected, the species for which a reliable transfer of single-photon responses is essential, i.e., crepuscular and nocturnal ones, tended to have both higher gain of phototransduction and somewhat bigger, more prolonged voltage bumps (Fig. 2a, c).

However, it can be seen from the comparison of group-average current and voltage bumps (Fig. 2a, c) that the pattern of amplitudes and kinetics of the voltage bumps does not generally match that of the current bumps. This can only be due to variation in the extent of membrane gain and low-pass filtering caused by differing input conductances and dissimilar *C*_m_ values (Table 1).

**Fig. 3:**
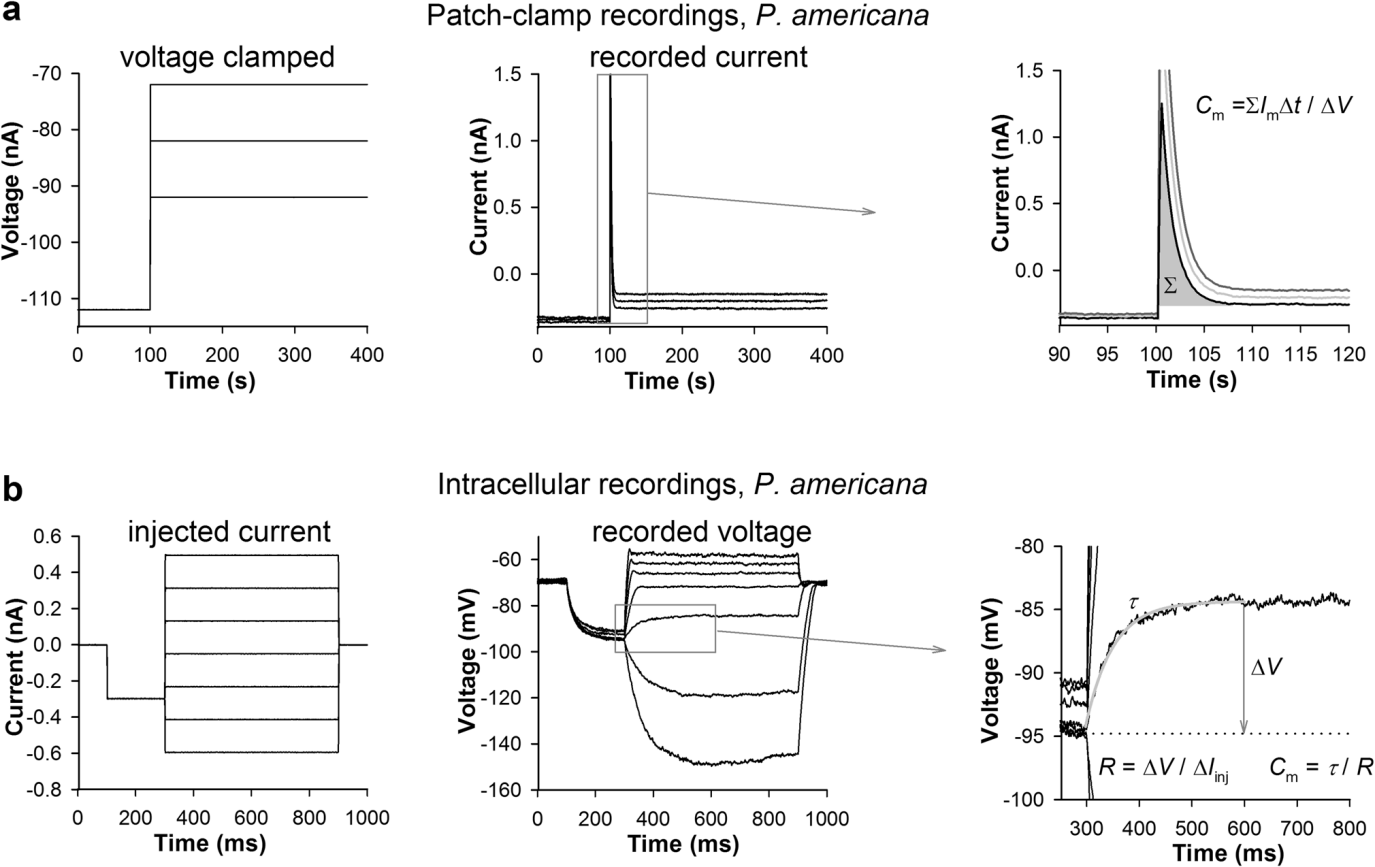
Evaluation of membrane capacitance. **a** In the patch-clamp experiments, *C*_m_ was obtained from current responses to voltage steps in the passive membrane voltage range in the absence of series resistance and capacitance compensations (left panel); current responses to three steps are shown in the center, with the magnified initial transients shown in the right panel. *C*_m_ was calculated by first integrating the initial capacitive transient (shaded area) by summing the products of the momentary current (*I*_m_) and the sampling interval (∆t) within the integration time range, and then dividing it with the voltage step amplitude (20 mV for the first step). **b** In the intracellular recording experiments, *C*_m_ was obtained from voltage responses to current steps (left panel); voltage responses are shown in the center, and the magnified responses to the right. *C*_m_ was calculated by first fitting the rising phase of a step response with a single exponential function (gray trace) to obtain membrane time constant (τ) and then dividing it with the membrane resistance (*R*); *R* was obtained by dividing the voltage response amplitude (∆*V*) after it settled by the injected current (∆*I*_inj_), which equaled 70 pA for this trace

Although we obtained group-average estimates of input conductance for all species using current measurements in voltage-clamp recordings, these estimates are indirect and do not take into account changes in membrane conductance *during* the elementary response. Therefore, we focused on the more reliable *C*_m_ values. Figure 3 shows how we measured photoreceptor capacitance in the patch-clamp (Fig. 3a) and intracellular recording experiments (Fig. 3b, also see “Methods”).

**Fig. 4:**
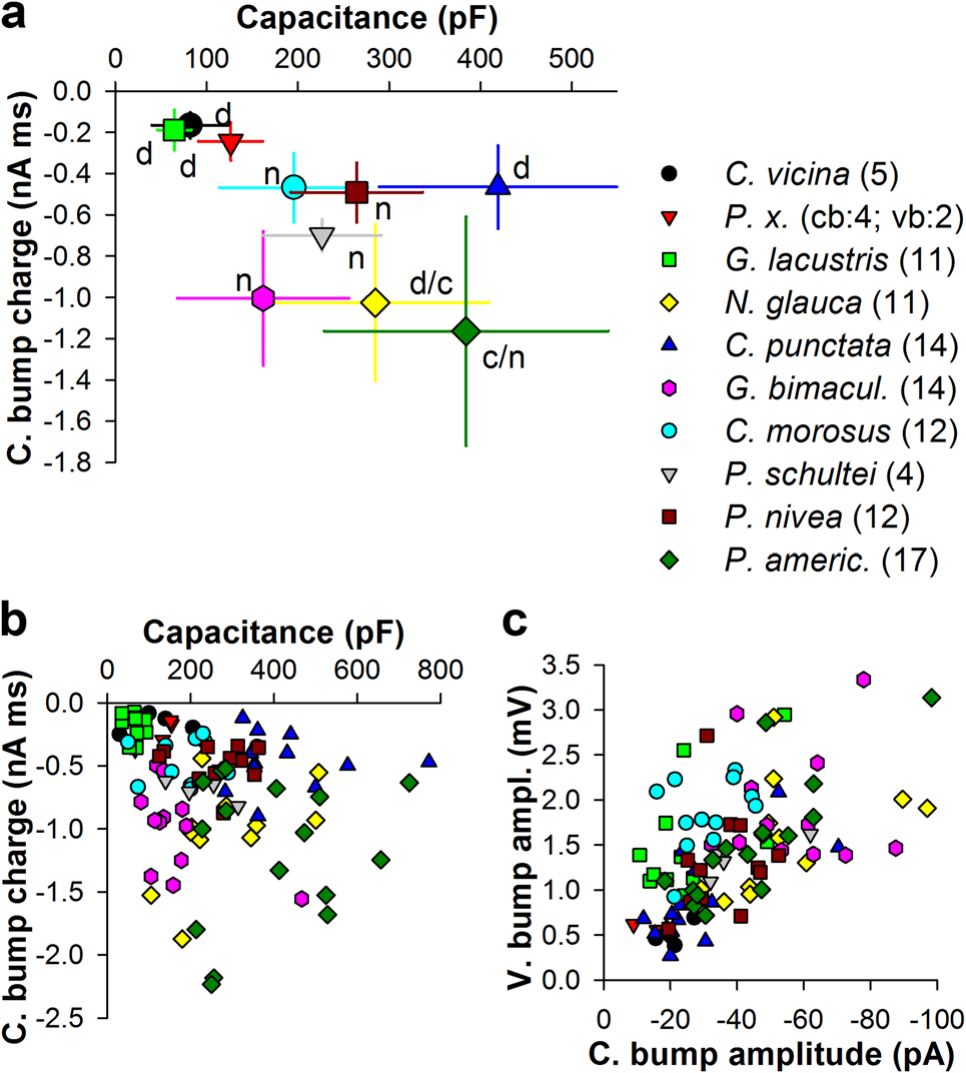
Correlations between quantum bump parameters and membrane capacitance. **a** Dependence of the group-average current bump integrals (charge) on *C*_m_; error bars denote s.d.; “n” stands for nocturnal, “d” diurnal, and “c” crepuscular lifestyles of the animals. **b** Dependence of mean current bump integrals on *C*_m_. **c** Dependence of mean voltage bump amplitudes on mean current bump amplitudes. All associated correlation coefficients are provided both in “Results” and in Table 2

Figure 4a shows a dependence of the group-average values of the total charge during the current bump on the group-average *C*_m_. The correlation was statistically significant (*ρ* = − 0.65, *P* = 0.038). A similar but smaller correlation was found when all data were pooled together (*ρ* = − 0.3, *P* = 0.003, Fig. 4b). Figure 4c shows the relationship between mean current and voltage bump amplitudes. Voltage bump amplitude increased linearly as current bump increased (Fig. 4c: *ρ* = − 0.59, *P* < 10^−6^; for the group-average data: *ρ* = − 0.76, *P* = 0.009).

### Signaling by voltage bumps

We hypothesized that the correlations in Fig. 4a, b represent an important evolutionary compensation aimed to alleviate the effects of low-pass filtering on the voltage signal. As high *C*_m_ associated with more sensitive photoreceptors of nocturnal insects dampens the voltage bump, increasing the gain of phototransduction might be instrumental to restore a voltage signal with sufficiently high SNR for vision in the near dark. However, to evaluate the SNR associated with single-photon responses and its dependence on the photoreceptor size, the properties of the voltage noise in the dark and surrounding the voltage bump responses must also be investigated. The background voltage noise consists of the genuine membrane voltage noise caused by various molecular fluctuations, such as the stochastic ion channel opening or closing, and instrumental voltage noise due to the external interference during signal acquisition and processing. Although the two components are inseparable, the prominent differences in voltage noise found between different species under the same experimental conditions, as we report next, suggest that the instrumental noise contribution to the voltage noise is minor. Therefore, below we address the voltage noise as the “membrane noise”.

Due to the high variability in *C*_m_ within the majority of studied species, and the expected non-specificity of *C*_m_ effects on both the light-induced voltage signals and membrane noise, in the following analysis we pooled all the data from all species together and looked for general trends. However, because different sample sizes skew correlations based on such combined plots, we also calculated correlation coefficients for the group-average data. All correlation coefficients are provided in Table 2.

**Table 2:** Spearman’s rank order correlation coefficients for correlations between electrophysiological parameters obtained in patch-clamp experiments

Parameter	*C*_m_, pF		Voltage bump amplitude, mV		Voltage bump integral, mV × ms	
	Pooled data	Group average	Pooled data	Group average	Pooled data	Group average
Current bump, pA	− 0.05 (0.63)	− 0.49 (0.14)	− 0.6 (< 10^−6^)	− 0.37 (0.002)	− 0.55 (0.09)	− 0.76 (0.009)
Current bump integral, pA.ms	− 0.3 (0.003)	− 0.65 (0.04)	− 0.46 (< 10^−5^)	− 0.41 (< 10^−4^)	− 0.64 (0.043)	− 0.67 (0.03)
Noise s.d., mV	− 0.69 (< 10^−6^)	− 0.9 (< 10^−6^)				
Voltage bump amplitude, mV	− 0.31 (0.002)	− 0.45* (0.23)				
Voltage bump integral, mV.ms	0.28 (0.005)	0.6 (0.06)				
SNR^1^	0.35 (0.0003)	0.84 (< 10^−6^)	0.5 (< 10^−6^)	0.26 (0.44)	0.66 (< 10^−6^)	0.67 (0.03)
SNR^2^	0.25 (0.01)	0.75 (0.01)	0.59 (< 10^−6^)	0.35 (0.31)	0.68 (< 10^−6^)	0.78 (0.005)

Numbers in parentheses denote *P* values. For all correlations, the number of data points was ≥ 95 for the pooled data and 10 for the group-average data

*This correlation was calculated with *C. vicina* and *P. xuthus* data excluded

^a^SNR was obtained by dividing the mean voltage bump amplitude by the mean noise event amplitude; mean noise event amplitudes are not provided because the metrics is arbitrary and proportional to noise s.d. values

^b^SNR was obtained by dividing the mean voltage bump amplitude by noise s.d

**Fig. 5:**
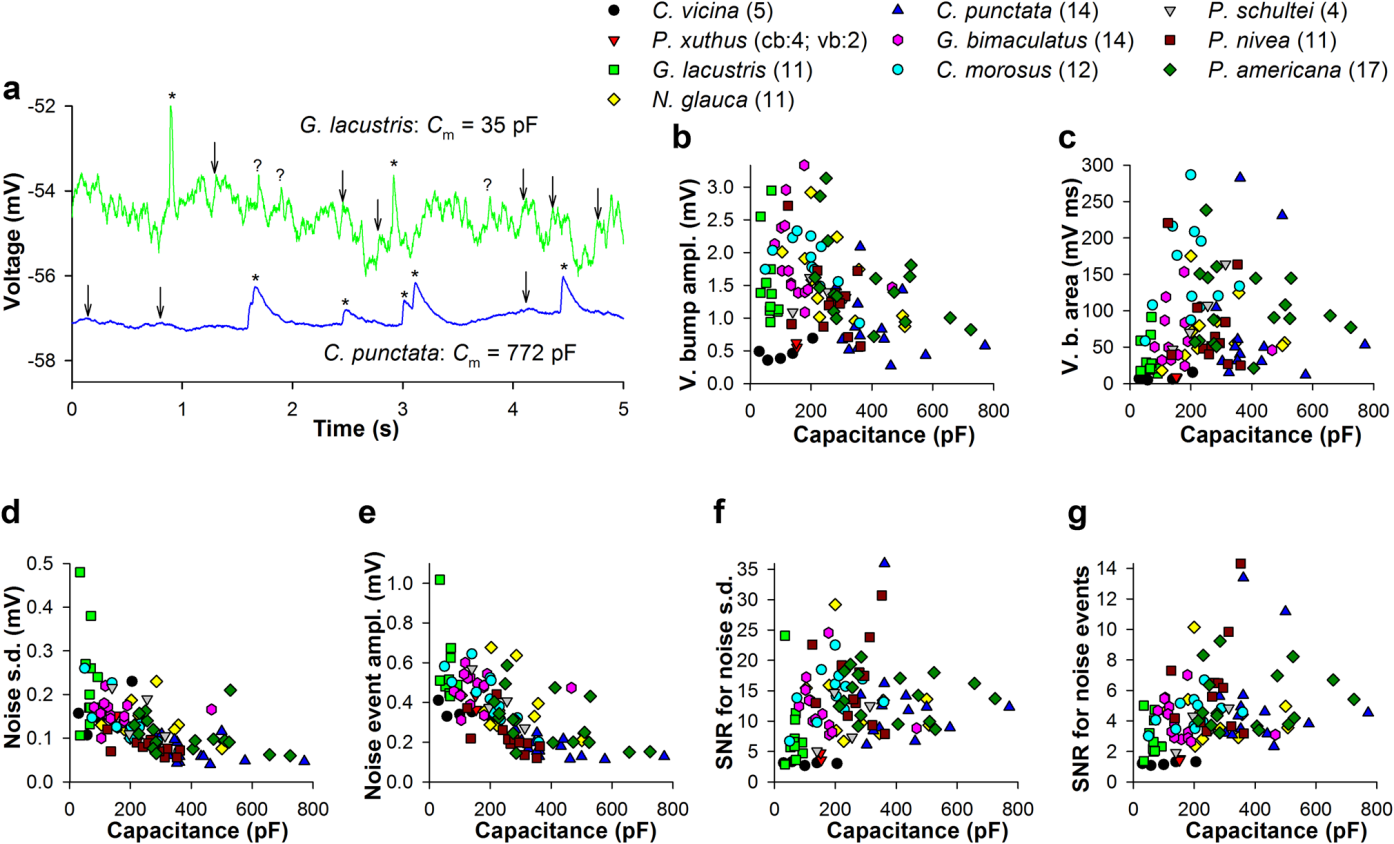
Signal and noise analysis. **a** 5-s fragments of voltage recordings from a small *G. lacustris* photoreceptor (green) and a large *C. punctata* photoreceptor (blue); cells were stimulated by very dim light; asterisks denote unambiguous single or double voltage bumps, question marks fast but small bump-like events, and arrows the largest noise events. Notice that even the largest noise events are very small and slow in the *C. punctata* recording. **b** Dependence of mean voltage bump amplitudes on *C*_m_; color coding for symbols is consistent for all panels; *n* is the number of cells; the differences in *n* between Figs. 4 and 5 are due to the absence of *C*_m_ measurements for some cells. **c** Dependence of mean voltage bump integrals on *C*_m_. **d** Dependence of membrane noise s.d. on *C*_m_. **e** Dependence of the largest membrane noise event amplitudes on *C*_m_. **f, g** Dependencies of SNR on *C*_m_; to obtain SNR for noise s.d. **f**, data from **b** were divided by data from **d**; to obtain SNR for noise events **g**, data from **b** were divided by data from **e**. All correlation coefficients are provided in Table 2

Figure 5a illustrates the differences in voltage bumps and membrane noise for photoreceptors with either very small (*G. lacustris*, 35 pF) or very high *C*_m_ (*C. punctata*, 772 pF) values. In the figure, apparent voltage bumps are marked with asterisks, possible voltage bumps with question marks, and the largest and fastest noise events with arrows. It can be seen that the *G. lacustris* recording contains many fast and high-amplitude noise events, which, if transmitted across the synapse, could interfere with upstream processing of the voltage bump signal from this photoreceptor. In contrast, the *C. punctata* recording is dominated by unambiguous voltage bumps, which, while characterized by relatively low amplitudes, are much faster and larger than any noise event present.

The calculation of SNR for voltage bumps raises the question of what constitutes a noise event. Depending on the voltage dependence of the presynaptic Ca^2+^ channels and the value of resting potential, the background membrane noise might continuously modulate the vesicle release rate at the first visual synapse, producing false signals (Juusola et al. 1995, 1996). In such circumstances, the strength of the vesicle release event in response to the true voltage signal would be proportional to both the amplitude *and* the duration of the voltage bump, i.e., to the area under the voltage bump curve. If, however, the situation in the nocturnal insects is different and some form of signal threshold is required, then only the largest of the noise events would trigger the vesicle releases at the terminal. Therefore, to account for all possible scenarios, we used several estimators for voltage bump signal and noise.

The mean bump amplitude and the total area under the voltage bump were used as signal estimators; in each cell, 10–30 bumps were selected randomly to yield the mean bump, the amplitude and the area of which was then used in correlations shown in Fig. 5. Figure 5b shows the dependence of mean voltage bump amplitudes on photoreceptor capacitance. Overall, a weak negative correlation was found, consistent with the attenuating effect of *C*_m_ on the voltage signal amplitude. If, however, the correlation was evaluated while excluding *C. vicina* and *P. xuthus* data characterized by very high input conductances and thus very low membrane gains (Table 1), the correlation would become substantially stronger (*ρ* = − 0.48, *P* < 10^−5^, Table 2). In contrast to voltage bump amplitudes, the total voltage bump signal as measured by the area under the mean voltage bump increased with *C*_m_ (Fig. 5c: *ρ* = 0.28, *P* = 0.005; for the group-average data: *ρ* = 0.6, *P* = 0.06).

For the noise, the first estimator was standard deviation calculated using relatively short fragments (100–1000 ms) of background membrane potential recorded either in the dark or obtained from intervals between voltage bumps during continuous low-intensity light stimulation. The noise s.d. decreased strongly with the increasing *C*_m_ (Fig. 5d; Table 2). The second estimator was the mean amplitude of the *largest depolarizing membrane noise events* (henceforth: noise events), such as those marked with arrows in Fig. 5a. The number of such events selected in each cell *matched the number of voltage bumps* used to evaluate the signal. The amplitudes of the noise events decreased strongly with *C*_m_ (Fig. 5e: *ρ* = − 0.71, *P* < 10^−6^). As expected, the two noise estimators correlated strongly positively, with *ρ* of 0.85 (*P* < 10^−6^, *n* = 103).

It can be seen from the comparison of plots in Fig. 5b, d, e that the estimators of noise decreased stronger with *C*_m_ than the estimators of signal, implying that SNR must increase as *C*_m_ increases. Indeed, the SNRs for voltage bumps calculated as the mean bump amplitude divided by either noise s.d. or the mean noise event amplitude increased progressively with *C*_m_ (Fig. 5f, g; Table 2). These findings indicate that increased *C*_m_ facilitates processing and transfer of voltage bumps by disproportionally reducing noise.

It should be noted that neither of our noise estimates are compelling measures of membrane noise: the standard deviation is an average that disregards both the kinetics and polarity of baseline changes; whereas the depolarizing membrane noise events might represent distorted bumps or signals from the neighboring cells. However, together these two measures provide a useful range to estimate SNR of the elementary signal.

**Fig. 6:**
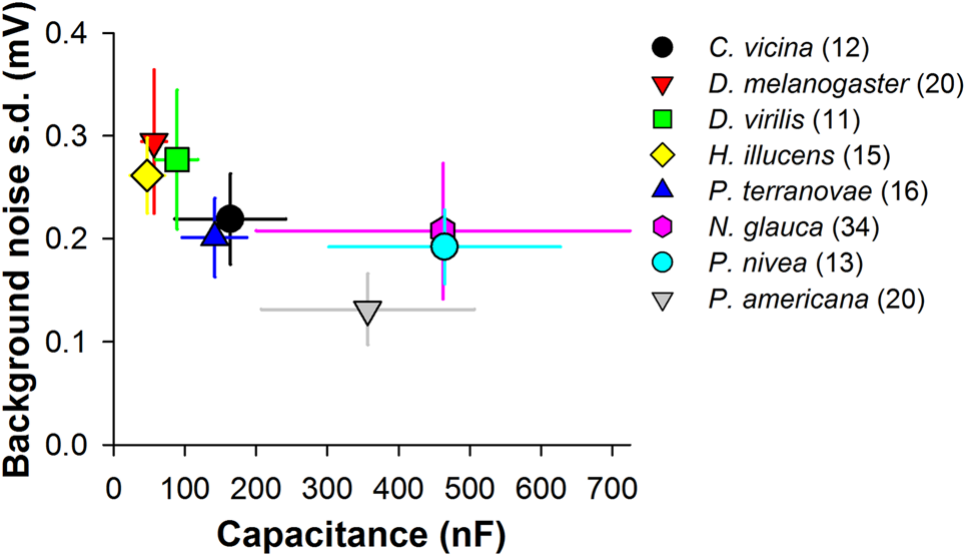
Background noise in the dark during intracellular recordings and *C*_m_. Scatter plot shows dependence of voltage noise in the dark on membrane capacitance for eight insect species. *C*_m_ values were determined as described in “Methods”; error bars denote s.d. To obtain noise estimates, 100 ms fragments of voltage recordings in the dark were de-trended, low-pass filtered using a 3 dB frequency of 200 Hz, and their s.d. values calculated; as result the noise s.d. values covered the range of 10–200 Hz

We also investigated if the trends found in patch-clamp experiments can be observed in intracellular recordings. We examined background voltage noise levels in recordings from eight species: the flies *C. vicina*, *Drosophila melanogaster*, *Drosophila virilis*, *Hermetia illucens*, and *Protophormia terraenovae*, water boatman *N. glauca*, and cockroaches *P. nivea* and *P. americana*. Consistently with the patch-clamp results, the noise decreased as *C*_m_ increased (Fig. 6: *ρ* = − 0.76, *P* = 0.02).

### Morphological correlates

**Fig. 7:**
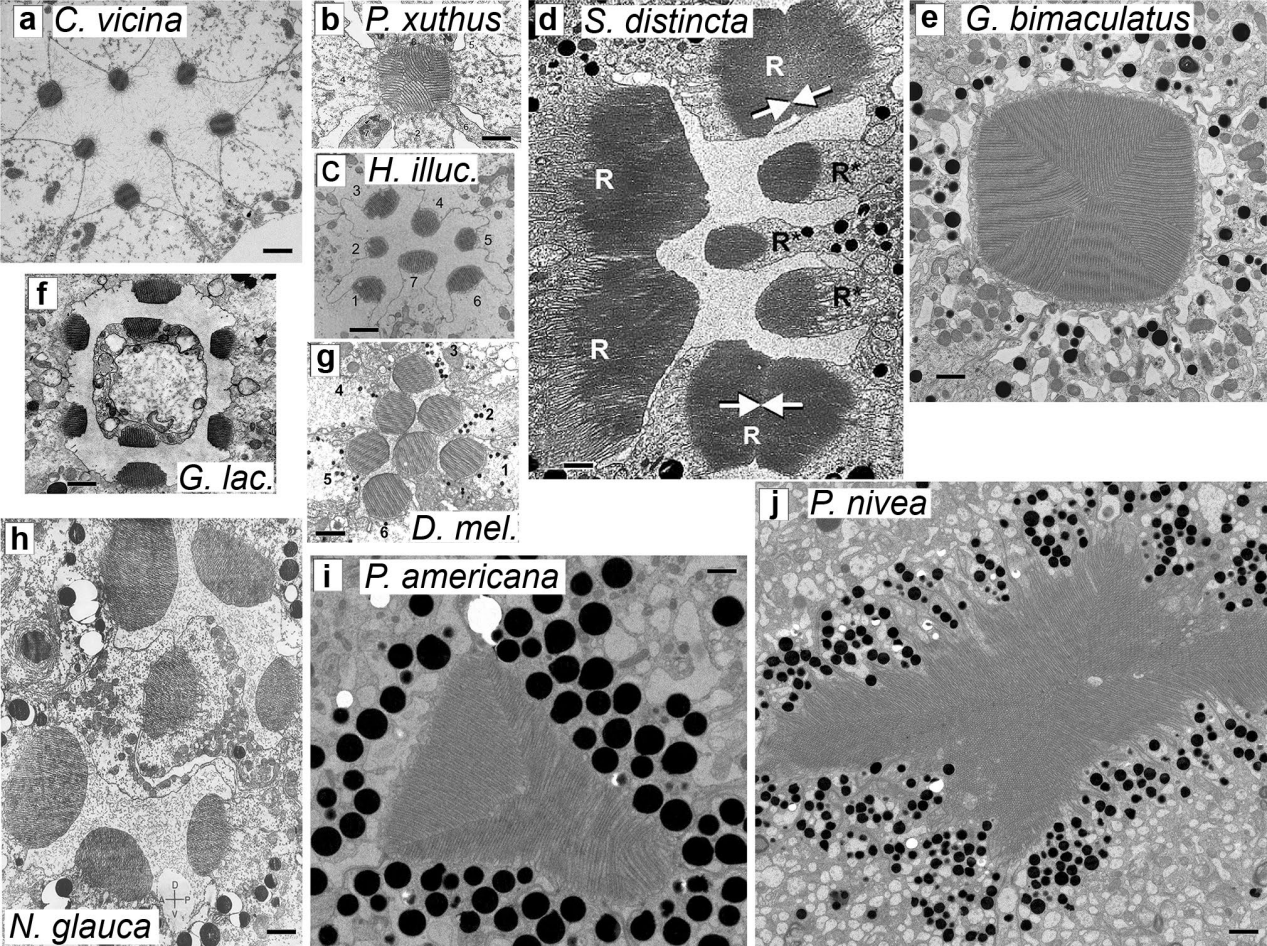
Micrographs of rhabdoms. High-resolution EM images of cross sections in the distal part of the retina are shown for: **a***C. vicina*; **b***P. xuthus* (adapted with permission from (Arikawa and Stavenga 1997)); **c***H. illucens* (adapted with permission from (Oonincx et al. 2016)); **d***S. distincta* as a proxy for *C. punctata* (adapted with permission from (Fischer et al. 2000)), notice two bipartite rhabdomeres formed by the same cell (arrows); **e***G. bimaculatus* (adapted with permission from (Meyer-Rochow et al. 2002)); **f***G. lacustris* (adapted with permission from (Schneider and Langer 1969)); **g***D. melanogaster* (adapted with permission from (Labhart and Meyer 1999)); **h** *N. glauca* (adapted with permission from (Horridge 1968)); **i***P. americana*; and **j***P. nivea*. Some images were edited for clarity. Scale bars are 1 µm

Because electrophysiological measurements of *C*_m_ in the highly compartmentalized microvillar photoreceptors often raise questions about their validity due to potential signal attenuation in the microvilli, it was necessary to correlate *C*_m_ values with the morphological data. Figure 7 shows high-resolution images of transverse sections of the distal parts of ommatidia from ten insect species, nine of which were used in this study, while the tenth, *Sigara distincta*, is a close relative of *C. punctata*, for which no high-resolution images could be found in literature. We were also unable to find EM images of stick insects’ retinas. It can be seen that there is a dramatic variation in the size of the rhabdomeres, with the smallest ones found in the diurnal *C. vicina*, *P. xuthus*, *H. illucens*, and *G. lacustris* (Fig. 7a–c, f), and also in the small crepuscular/diurnal fly *D. melanogaster* (Fig. 7g). Rhabdomeres of these species are characterized by a relatively small number of short microvilli (Table 3). In contrast, the rhabdomeres of the nocturnal *G. bimaculatus, P. americana*, and *P. nivea* (Fig. 7e–j) are very large and contain numerous relatively long microvilli. The rhabdomeres of the two aquatic species are also large, and systematically and conspicuously vary in size (Fig. 7d, h), which is probably a prerequisite for effective functioning under disparate illumination conditions (Immonen et al. 2014a).

**Table 3:** Electrophysiological and morphological estimates of rhabdomere size for the species in Fig. 7

Species	*C*_m_, pF	Max. microvillus length, µm	Number of microvilli per cross-section	Microvillus spacing, nm	Approx. rhabdomere/ ommatidium length, µm	References
*C. vicina*	pc: 82 ± 42 ir: 164 ± 80	0.8 ± 0.1	19.8 ± 1.7	37.0 ± 1.0	300	(Schmitt et al. 2005)
*D. melanogaster*	pc: 55 ir: 57 ± 17	1.2 ± 0.1	34.3 ± 4.3	52.2 ± 7.0	85	(Pollock et al. 1990); (Chevesich et al. 1997); (Labhart and Meyer 1999); (Frolov et al. 2016)
*H. illucens*	ir: 48 ± 24	0.8 ± 0.1	16.2 ± 3.8	59.0 ± 4.5	350	(Oonincx et al. 2016)
*P. xuthus*	pc: 127 ± 35	1.0 ± 0.2	15.1 ± 2.5	88.5 ± 5.3	440^c^	(Arikawa and Stavenga 1997); (Arikawa et al. 1999)
*G. lacustris*	pc: 65 ± 19	1.1 ± 0.3	32.4 ± 10.2	59.1 ± 2.4	115	(Schneider and Langer 1969); (Fischer et al. 2000); (Frolov and Weckstrom 2014)
*N. glauca*	pc: 285 ± 124 ir: 462 ± 262	2.1 ± 0.6	51.9 ± 10.2	59.8 ± 1.4	110	(Horridge 1968); (Fischer et al. 2000); (Immonen et al. 2014a)
*C. punctata*	pc: 419 ± 130	3.1 ± 0.8^a^	94.7 ± 3.8^b^	n/a	140	(Fischer et al. 2000); (Frolov 2015);
*G. bimaculatus*	pc: 162 ± 94	2.8 ± 0.6	33.1 ± 7.9	92.0 ± 6.2	240^c^	(Meyer-Rochow et al. 2002); (Sakura et al. 2003); (Henze et al. 2012); (Frolov et al. 2014)
*P. nivea*	pc: 265 ± 72 ir: 465 ± 162	3.0 ± 0.4	90.0 ± 33.1	70.0 ± 6.0	114^c^	(Frolov et al. 2017)
*P. americana*	pc: 384 ± 156 ir: 357 ± 149	2.7 ± 0.6	70.9 ± 31.3	72.0 ± 7.0	182^c^	(Frolov et al. 2017)

Two group-average *C*_m_ values are provided whenever possible, from patch-clamp (pc) and intracellular recording (ir) experiments

Morphological measurements were made using micrographs both in Fig. 7 and in the articles cited. The number of data points for each mean was ≥ 5. The three microvillus-related measurements were made for each rhabdomere cross-sectional image separately and then averaged. The microvillus spacing was calculated as the width of the rhabdomere divided by the number of microvilli in the cross section; since the small but measurable distance between the neighboring microvilli (see e.g., (Frolov et al. 2017)) is not taken into the account, this metric slightly overestimates the actual mean diameter of the microvillus. For the blowfly, the microvillus measurements were made using our own micrographs, whereas the length of the ommatidium was inferred from the reference provided. In the open-rhabdom ommatidia, only peripheral rhabdomeres were used in measurements

^a^From Fig. 7d

^b^Data from *S. distincta*, assuming a 60 nm microvillus spacing

^c^These ommatidia are either two-tiered, or individual rhabdomeres contribute differently to the rhabdom at different levels

**Fig. 8:**
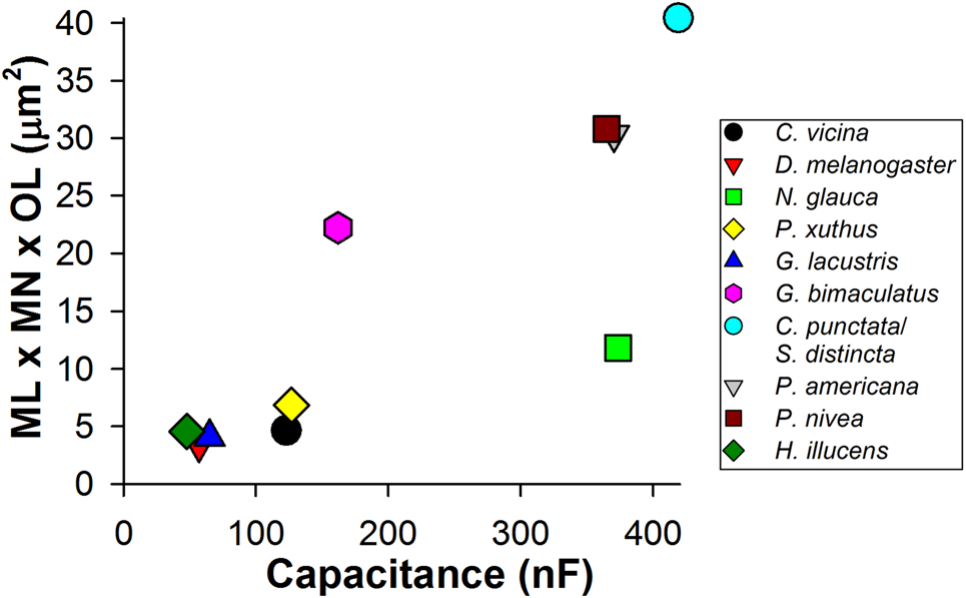
Rhabdomere membrane and *C*_m_ estimates. Light-sensitive membrane area estimates were obtained by multiplying the parameters describing photoreceptor morphology from Table 3: the maximal microvillus length (ML), approximated by the microvillus spacing value, the number of microvilli per rhabdomere cross section (MN), and the length of the ommatidium (OL). *C*_m_ values in the scatter plot were taken from Table 3 as averages of the patch-clamp and intracellular recording (where available) data. The SROCC was 0.88 (*P* < 10^−6^)

A measure proportional to the area of the light-sensitive membrane can be obtained by multiplying the microvillus length, the microvillus diameter, the number of microvilli per cross section, and the number of microvilli per length of the rhabdomere. This reduces to the product of the microvillus length, the number of microvilli per cross section, and the rhabdomere length. When such measures were calculated and plotted against the corresponding *C*_m_ values, a strong positive correlation has emerged (Fig. 8), suggesting that our electrophysiological estimates are indeed proportional to the cell membrane areas. However, it should be noted that such approximations take into account neither the rest of the photoreceptor plasma membrane, nor the variation in the size of the rhabdomere along the length of the cell (especially in two-tiered rhabdoms) nor the large variation in the length of microvilli within the rhabdomere.

## Discussion

**Fig. 9:**
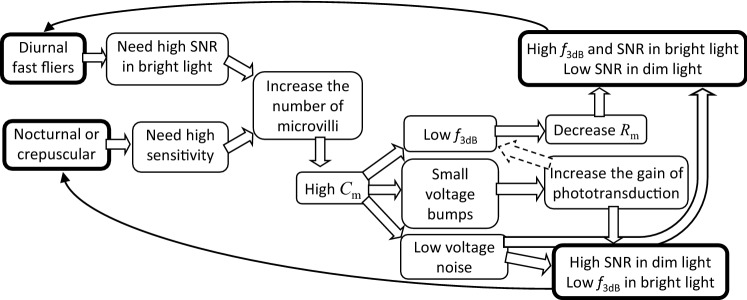
A scheme of proposed evolution of the elementary voltage responses. The flowchart suggests how natural selection under drastically different conditions, in the dark or during flight in daylight, might shape the morphological and electrophysiological properties of microvillar photoreceptors

In this work, we investigated the properties of elementary electrical responses of photoreceptors from ten insect species characterized by different visual system morphologies, lifestyles and behaviors. Previous comparative studies provided evidence of profound electrophysiological differences between photoreceptors of insects occupying different ecological niches (Laughlin and Weckström 1993; Weckstrom and Laughlin 1995; Frederiksen et al. 2008; Frolov 2016; Honkanen et al. 2017). In particular, two evolutionary developments stand out: a specialization for fast flight and aerial maneuvering in daylight, and a specialization for vision in the near dark (Fig. 9). The former manifests in the unsurpassed temporal resolution and very high SNR in photoreceptors of blowflies (Niven et al. 2007). The latter allows reliable processing of rare single-photon absorption events (Honkanen et al. 2017).

Despite the different end effects, both high SNR in bright light and high absolute sensitivity in dim light are associated with enlarged rhabdomeres (Frolov 2016), which leads to increased whole-cell capacitance (Table 3; Fig. 9). In the case of diurnal fliers, the increased rhabdom sizes appear to be an adaptation countering the inactivation of the microvilli in bright light (photo-bleaching). Due to their refractoriness, the total number of microvilli needs to be high enough to ensure the presence at all times of a functional pool of sampling units (Song and Juusola 2014). Such a ready for activation fraction can be on the order of a percentage point, but if the overall number of microvilli is over 100 000, it might be sufficient to mediate graded signal transfer without excessive photon shot noise.

Because *C*_m_ non-linearly slows and attenuates fast and often small voltage changes in daylight and thus limits the temporal resolution, which is unacceptable for fast fliers, an increased *C*_m_ is compensated for by increased sustained K^+^ conductances, which in turn leads to a high metabolic cost of information (Niven et al. 2007; Niven and Laughlin 2008). In contrast, reliable transfer of single-photon responses, the voltage bumps, is necessary for vision in very dim environments. One would expect that photoreceptors of nocturnal insects should be characterized by a higher SNR for voltage bumps than photoreceptors of diurnal insects. However, high *C*_m_ lowers the amplitude of voltage bumps. As we have shown here, this is compensated for by the increased gain of phototransduction so that species active in the dark have larger and more prolonged current bumps (Figs. 2, 4), and by the reduced background voltage noise (Figs. 5, 6). However, an increase and prolongation of the current bump can worsen the temporal resolution, because photoreceptor bandwidth ultimately depends on the duration of the elementary response.

The three main findings of this study were: (1) the increase of the gain of phototransduction with *C*_m_, (2) the decrease in membrane noise level with increasing *C*_m_, and (3) the accompanying increase in the SNR of voltage bumps.

As discussed above, fast diurnal species were characterized by small current and voltage bumps, while the nocturnal/crepuscular ones by large elementary responses. We propose that the associated dependence of the current bump signal on *C*_m_ represents an evolutionary strategy that facilitates signaling at the level of isolated voltage bumps, i.e., in the near dark (Fig. 9). But how can an animal change its *basic* gain of phototransduction? Two strategies are suggested by comparative studies: a change in the average size of the microvillus, and a change in the composition of light-activated channels. The lengths of the microvilli appear to be larger in general in the nocturnal than in diurnal insects (Figs. 7, 8; and Table 3). This alone can increase the current bump if the densities of light-activated channels in the microvillus remain approximately constant. The second scenario involves qualitative changes in the composition of light-activated channels. Photoreceptors in *D. melanogaster* normally express TRP as the predominant channel type (~ 90%) (Hardie and Minke 1992; Niemeyer et al. 1996; Reuss et al. 1997; Leung et al. 2000). TRP is characterized by a relatively small unitary conductance in vitro, and the current bump in the fly is accordingly quite small, less than 10 pA on average under the same recording conditions as in our experiments (Reuss et al. 1997; Henderson et al. 2000). However, flies raised in the dark express elevated levels of TRPL channels characterized by higher unitary conductance (Reuss et al. 1997; Bahner et al. 2002). Consistent with this, the nocturnal *P. americana* expresses mainly TRPL channels and its current bumps are large (Fig. 2a) (Saari et al. 2017), due to bigger contribution of Na^+^ to the current. As the microvilli in the nocturnal species are generally longer than in the diurnal ones (Fig. 7; Table 3), it appears that both gain augmentation strategies might have been used in evolution.

Interestingly, we also found large inter-species variability in the diameter of the microvillus as approximated by the microvillus spacing (Table 3). The microvillus diameter in the fast-flying blowfly *C. vicina* (and also houseflies such as *Musca domestica* (Wunderer et al. 1989)) was below 40 nm. It was larger, from 50 to 60 nm, in the slower flies and in the water bugs, and still larger in the cockroaches, ca. 70 nm (Table 3). The widest microvilli, ca. 90 nm in diameter, can be found in the cricket *G. bimaculatus* and, surprisingly, in the fast diurnal butterfly *P. xuthus* (Table 3). Other butterflies possess similarly wide microvilli [see, e.g., (Meyer-Rochow et al. 2002)]. Whether these differences in the microvillus diameter have functional consequences is not known.

Because photoreceptors of the same spectral class in the same retina can vary strongly in *C*_m_ (Frolov 2016, 2019), a question arises whether the correlation between the group-average *C*_m_ and the group-average quantum bump gain [measured as the current bump integral (Fig. 4a)] represents an evolutionary or an ontogenetic development. However, in our analyses of separate species we found no significant correlations between *C*_m_ and mean current bump integrals (data not shown). As another example, when the group-average *C*_m_ was reduced by about 2.5 times in *P. americana* by chronic exposure to daylight, current bumps did not change significantly (Frolov et al. 2018).

What causes the decrease in membrane noise as *C*_m_ increases? A likely reason is that as low-pass filtering slows, attenuates and spreads a fast voltage change, the numerous membrane noise events are summed and averaged out, whereas the rare voltage bumps evoked in dim light remain isolated. It should be noted that although we observed similar trends in patch-clamp and intracellular recording experiments, the difference in the levels of background noise between high and low *C*_m_ species was somewhat smaller in intracellular recordings, probably due to the higher level of instrumental noise associated with using high-resistance microelectrodes.

We evaluated SNR directly using two approaches, by dividing voltage bump amplitudes with either mean noise s.d. values or noise event amplitudes. Both plots in Fig. 5f and g indicate that SNR increases with *C*_m_. However, this could be an underestimation of the true increase because, in contrast to voltage bump amplitudes that correlated negatively with *C*_m_, the strength of the total voltage signal actually increased as *C*_m_ grew (Fig. 5c; Table 2). However, we did not attempt to estimate the associated SNR because of the absence of an appropriate corresponding measure for noise.

There is still another observation pointing to the increase in SNR with increase in *C*_m_. A salient difference between voltage responses of small versus large photoreceptors is the disappearance of membrane noise events with relatively fast onset kinetics (Fig. 5a). As a consequence, over the entire *C*_m_ range, the voltage bumps represent the *fastest depolarizing voltage changes* in the photoreceptor. Both the voltage bump and noise events are slowed by the increased low-pass filtering as *C*_m_ grows but membrane noise appears to be suppressed more strongly, so that the ratio of onset depolarization rates (10–90% amplitude change rate) for the voltage bump and noise events increased with the size of the photoreceptor quite dramatically, from about two in *C. vicina* to over eight in *C. punctata* (data not shown). The difference in the rates of depolarization can probably affect the signal transfer at the synapse, because a faster depolarization might elicit a faster and more concerted opening of the presynaptic Ca^2+^ channels than a slower depolarization and result in a more robust surge of Ca^2+^ across the membrane and thus in an increased probability of vesicle release or a stronger change in the rate of continuous release.

## Conclusions

In this study, we discovered that the gain of phototransduction in microvillar photoreceptors depends on the group-average photoreceptor size, suggesting an evolutionary adaptation. Our results indicate that the photoreceptor size increases due to the growth of the rhabdomere, and most saliently in the species that do or can operate under near dark. We proposed here that the increased current bump helps to overcome the attenuating effect of *C*_m_ on the voltage signal, and thus improves the SNR. However, it appears that increased *C*_m_ is itself a useful adaptation as it strongly reduces the background voltage noise in the dark and thus decreases spurious signaling.

## Abbreviations

*C*_m_: Membrane capacitance, in picofarads (pF)
*f*_3dB_: Membrane corner frequency
*R*_m_: Membrane resistance
SNR: Signal-to-noise ratio
TRP: Transient receptor potential channel
TRPL: Transient receptor potential-like channel
